# IL-4 contributes to failure, and colludes with IL-10 to exacerbate *Leishmania donovani* infection following administration of a subcutaneous leishmanial antigen vaccine

**DOI:** 10.1186/1471-2180-14-8

**Published:** 2014-01-15

**Authors:** Sudipta Bhowmick, Rajesh Ravindran, Nahid Ali

**Affiliations:** 1 Current Address: Department of Zoology, Dr. Kanailal Bhattacharyya College, Dharmatala, Ramrajatala, Santragachi, Howrah 711104, India; 2 Current Address: Department of Pathology, Emory Vaccine Center, 954 Gatewood Road, Atlanta, GA 30329, USA; 3 Infectious Diseases and Immunology Division, Indian Institute of Chemical Biology, Kolkata, West Bengal, India

**Keywords:** *Leishmania donovani*, Alum, Saponin, IL-4, IL-10

## Abstract

**Background:**

Visceral leishmaniasis caused by the protozoan parasite *Leishmania donovani* complex is a potentially fatal disease if left untreated. Few treatment options exist and are toxic, costly and ineffective against resistant strains. Thus a safe and efficacious vaccine to combat this disease is needed. Previously, we reported that intraperitoneal administration of leishmanial antigens (LAg) entrapped in liposomes conferred protection to BALB/c mice against *L. donovani* challenge infection. However, this vaccine failed to protect mice when administered subcutaneously. We therefore evaluated whether formulation of LAg in combination with two commonly used human-compatible adjuvants, alum and saponin, could improve the protective efficacy of subcutaneously administered LAg, to a level comparable to that of the intraperitoneal liposomal vaccination.

**Results:**

Vaccine formulations of LAg with alum or saponin failed to reduce parasite burden in the liver, and alum + LAg immunized mice also failed to reduce parasite burden in the spleen. Interestingly, saponin + LAg vaccination actually resulted in an increased *L. donovani* parasitic load in the spleen following *L. donovani* challenge, suggesting this regimen exacerbates the infection. In contrast, mice immunized intraperitoneally with Lip + LAg demonstrated significant protection in both liver and spleen, as expected. Mechanistically, we found that failure of alum + LAg to protect mice was associated with elevated levels of IL-4, whereas both IL-4 and IL-10 levels were increased in saponin + LAg immunized mice. This outcome served to exacerbate *L. donovani* infection in the saponin + LAg group, despite a concurrent increase in proinflammatory IFN-γ production. On the contrary, protection against *L. donovani* challenge in Lip + LAg immunized mice was associated with elevated levels of IFN-γ in conjunction with low levels of IL-4 and IL-10 production.

**Conclusions:**

These findings indicate that elevated levels of IL-4 may contribute to LAg vaccine failure, whereas combined elevation of IL-4 together with IL-10 exacerbated the disease as observed in saponin + LAg immunized mice. In contrast, a robust IFN-γ response, in the absence of IL-4 and IL-10 production, was associated with protective immunity following administration of the Lip + LAg vaccine. Together these findings suggest that optimization of antigen/adjuvant formulations to minimize IL-4 and IL-10 induction may be helpful in the development of high efficacy vaccines targeting *Leishmania*.

## Background

Leishmaniasis is an important global public health problem with an estimated 350 million people at risk of infection. The disease is caused by parasites of the genus *Leishmania* and can be classified into three major forms based on their clinical manifestations. Whilst cutaneous leishmaniasis (CL) and mucocutaneous leishmaniasis (MCL) represent milder forms of the disease, visceral leishmaniasis (VL) is associated with a high mortality rate [1]. Currently, the available antileishmanial drugs are costly, toxic, induce severe side effects, and are ineffective against emerging drug resistant *Leishmania* strains. Therefore, the study and development of additional safe and effective vaccine regimens for clinical use remains critical.

The production of vaccines to combat leishmaniasis is increasingly reliant on subunit antigen constructs. Whilst defined antigens offer advantages in terms of safety, they are typically less immunogenic and require the addition of an adjuvant to be effective [2,3]. In our attempt to design a vaccine against VL we initiated studies with antigens of *Leishmania donovani* promastigotes (LAg) in association with liposomes as a vaccine delivery vehicle, as well as an adjuvant. Entrapment of LAg in liposomes led to remarkable levels of protection against *L. donovani* infection in hamsters and BALB/c mice when administered through the intraperitoneal route [4,5]. However, immunization via the subcutaneous route with the same liposomal vaccine failed to elicit protection [6]. This low efficacy following subcutaneous injection represents a critical barrier that currently limits the clinical applicability of a liposomal LAg subunit vaccine.

Whilst many adjuvants which are routinely used in laboratory animals are often incompatible for human use, alum has been licensed for human vaccines for decades and is still widely incorporated into new vaccine formulations currently in development [7]. In relation to leishmaniasis, alum has been used in combination with IL-12 and killed promastigotes, resulting in effective protection in a primate model of CL [8]. Furthermore, an alum-absorbed preparation of autoclaved *L. major* (alum-ALM) mixed with *Bacillus Calmette-Guerin* (BCG) protected Langur monkeys against VL [9]. Indeed, alum-ALM was found to be tolerable in healthy volunteers, whilst imparting minimal side-effects and conferring improved immunogenicity compared to preparations lacking the alum component [10]. These observations led to the use of this vaccine as an immunological stimulus for the treatment of patients with persistent post kala-azar dermal leishmaniasis (PKDL), where vaccine administration was shown to significantly improve the clinical outcome of PKDL lesions [11].

Saponin consists of natural glycosides of steroid or triterpene, which can activate the mammalian immune system, leading to significant interest in developing saponin as a vaccine adjuvant. Saponin has already been included as an adjuvant in clinical vaccine formulations against HIV and cancer [12]. Combined administration of saponin and fucose manose ligand (FML) antigen from *L. donovani* was additionally found to be protective against VL in both mice and dogs [13,14], and moreover the FML-vaccine was also effective in an immunotherapeutic context against the same disease [15,16]. Similarly the Leishmune® vaccine, composed of FML antigen with an increased concentration of saponin exhibited immunotherapeutic potential in dogs, reducing clinical symptoms following *L. chagasi* challenge [17]. There is therefore much hope for a saponin-adjuvanted leishmanial vaccine in veterinary and clinical research.

Alum and saponin are both approved for human use and have been widely applied in numerous clinical vaccine trials [7,12]. Therefore, in the present study we investigated the protective efficacy of LAg against *L. donovani* challenge in isolation, or in combination with either alum or saponin adjuvants administered through a subcutaneous route, as compared to the highly efficacious intraperitoneal route of lip + LAg administration in BALB/c mice.

## Results

### LAg immunization in combination with alum or saponin fails to reduce parasite burden, whereas a lip + LAg vaccine regimen induces protective immunity in the liver

**Figure 1:**
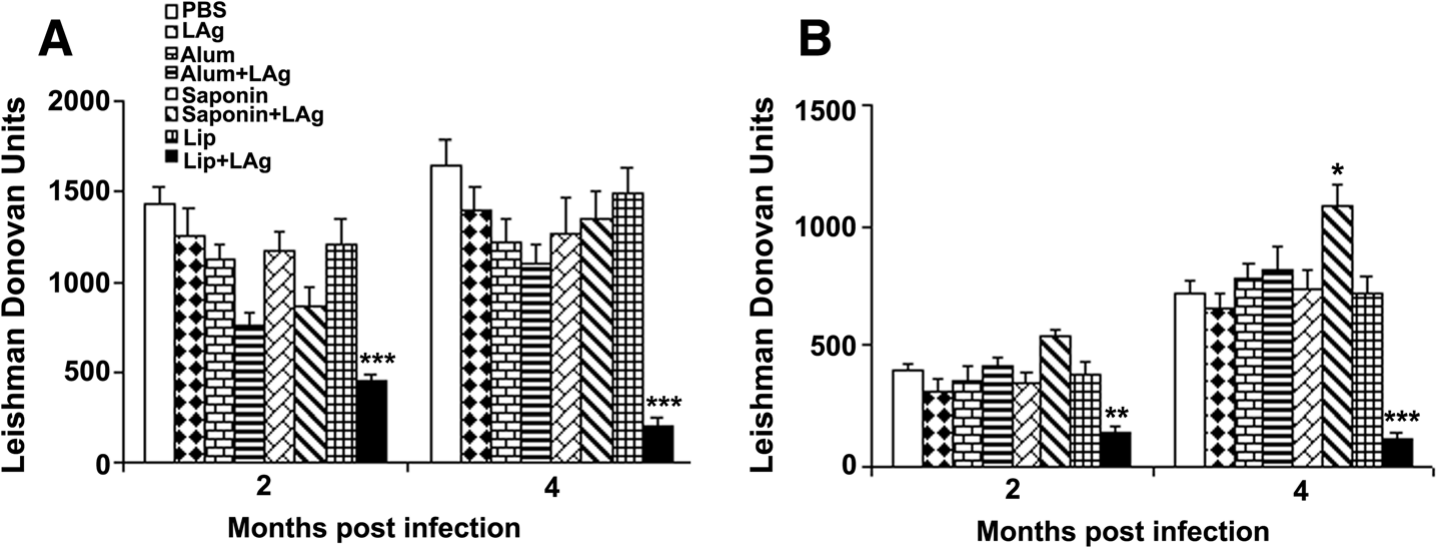
**Parasite burdens in vaccinated mice after *L. donovani *challenge infection.** BALB/c mice were vaccinated subcutaneously with PBS, LAg, alum, alum + LAg, saponin and saponin + LAg, or intraperitoneally with Lip and Lip + LAg. Ten days post-immunization, mice were challenged intravenously with 2.5 × 10^7^ promastigotes of *L. donovani*. Liver **(A)** and spleen **(B)** parasite burden was measured 2 and 4 months after challenge, and expressed as Leishman Donovan Units. Bars represent the mean ± SE of five individual mice per group, representative of two independent experiments. * *p* < 0.05, ** *p* < 0.01, *** *p* < 0.001 in comparison to PBS as well as free adjuvant immunized groups as assessed by a one-way ANOVA and Tukey’s multiple comparison test.

To determine the protective efficacy of LAg formulated in alum, saponin or liposomes, cohorts of naive BALB/c mice underwent a prime-boost immunization regimen with subcutaneously administered alum + LAg or saponin + LAg. As control, mice were administered with lip + LAg vaccine intraperitoneally, whereas negative control mice received PBS or adjuvant alone (subcutaneously). Mice were then challenged with *L. donovani* promastigotes 10 days after vaccination. Inoculation of BALB/c mice with *L. donovani* strain AG83 leads to progressive infection in the liver and spleen, corresponding with hepato- and splenomegaly [4,18]. We therefore evaluated the kinetics of increasing parasitic burden at 2 and 4 months after challenge, and the parasite loads in liver and spleen were quantitated as Leishman Donovan Units (Figure 1).

In the liver, we observed a trend of decreased parasitic load in both alum + LAg and saponin + LAg immunized mice as compared to PBS immunized control group, reaching statistical significance at 2 months postinfection (*p* < 0.05, Figure 1A). However, this effect was minor, and notably neither vaccine statistically improved the protective efficacy over immunization with adjuvant alone. Mice immunized with LAg alone also did not exhibit significantly reduced parasite load compared to controls, consistent with our earlier observation that free LAg administered subcutaneously did not influence parasite growth in the liver [6]. In contrast, significantly reduced parasite burden was seen following intraperitoneal immunization with lip + LAg as compared to both PBS and empty liposome immunized mice (*p* < 0.001) [4,6]. At 4 months postinfection both alum + LAg and saponin + LAg immunized mice failed to maintain the slight reduction in the parasite levels seen at the 2 month time point, instead demonstrating infection levels comparable to PBS and free adjuvant-immunized controls. In contrast, lip + LAg immunized animals maintained lower levels of parasite burden versus controls (*p* < 0.001).

### Immunization with alum + LAg fails to reduce splenic *L. donovani* burden whereas immunization with saponin + LAg exacerbates infection

In VL, the spleen acts as a reservoir for parasitic persistence, which is further associated with induction of host tolerance, and failure to clear the disease [4,5,18]. We therefore wished to monitor the effect of immunization with different LAg vaccine formulations on the splenic persistence of *L. donovani* following challenge. At 2 months postinfection, alum + LAg and saponin + LAg immunized cohorts both failed to control *L. donovani* infection in spleen, exhibiting parasite burden comparable to PBS and free adjuvant-immunized controls (Figure 1B). Failure to protect against infection in mice immunized with alum + LAg was also observed 4 months after infection. Contrary to our expectations, we observed significantly increased parasite burden in the spleen of mice immunized with saponin + LAg at the 4 month time point (*p* < 0.05) indicating this vaccine regimen exacerbated infection. In opposition, lip + LAg immunized mice showed a significant reduction in splenic parasite burden at 4 months post infection (*p* < 0.001 in comparison to PBS and free adjuvant-immunized controls), as expected [4].

### Induction of humoral response in immunized mice

**Figure 2:**
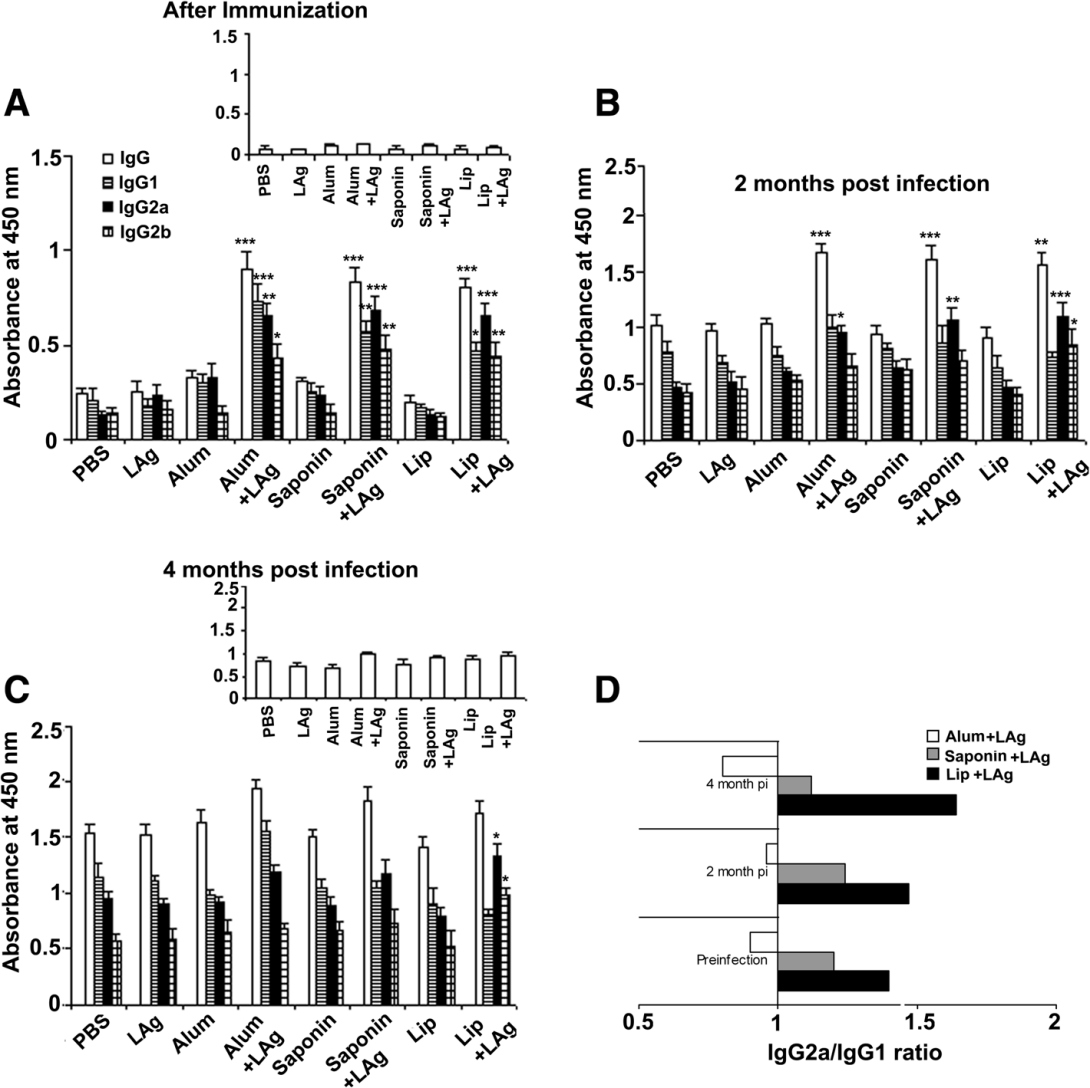
**Humoral response in vaccinated mice following immunization and *L. donovani *challenge infection.** Mice were immunized subcutaneously with PBS, LAg, alum, alum + LAg, saponin, saponin + LAg, or intraperitoneally with Lip and Lip + LAg. ELISA measurement of LAg-specific IgG, IgG1, IgG2a and IgG2b antibodies was performed on sera obtained from mice post-immunization **(A)**, 2 months **(B)** and 4 months **(C)** after challenge with *L. donovani.* The *insets* in **(A)** and **(C)** show antibody levels to the non-leishmanial control antigen OVA. Each sample was examined in duplicate. The results are shown as the mean absorbance values ± SE of five individual mice per group, representative of two independent experiments with similar results. IgG2a/IgG1 ratio in alum + LAg, saponin + LAg and Lip + LAg immunized mice **(D)** preinfection, 2 months and 4 months postinfection (pi). * *p* < 0.05, ** *p* < 0.01, *** *p* < 0.001 in comparison to PBS as well as free adjuvant immunized groups as assessed by one-way ANOVA and Tukey’s multiple comparison test.

VL is characterized by polyclonal antibody response, which helps to establish and maintain infection [19] and may even lead to disease exacerbation [20]. Thus it was of interest to investigate whether a specific/nonspecific antibody response plays a role in dictating vaccine efficacy. Sera were collected from immunized mice before *L. donovani* challenge, after 2 and 4 months of infection and assayed for LAg specific total IgG, and its isotypes IgG1, IgG2a and IgG2b. At 10 days post-vaccination, mice immunized with alum + LAg, saponin + LAg and lip + LAg induced significantly higher levels of LAg-specific IgG, and its isotypes IgG1, IgG2a and IgG2b in comparison to PBS as well as free adjuvant-immunized controls (Figure 2A, *p* < 0.05). IgG2a and IgG1 are surrogate markers for Th1 and Th2 responses, respectively [21], and both lip + LAg (1.40) and saponin + LAg (1.2) immunized mice showed a high IgG2a:IgG1 ratio that was suggestive of a Th1 bias, whereas the IgG2a:IgG1 ratio in alum + LAg immunized mice (0.90) revealed a skewing towards Th2 (Figure 2D). As control for the specificity of the response, serum antibody levels to a nonleishmanial antigen OVA were also assessed, and we observed minimal reactivity in all experimental conditions at 10 days post-vaccination (Figure 2A, *inset*).

After 2 months post- *L. donovani* infection, the levels of IgG increased further in alum + LAg and saponin + LAg immunized mice, differing significantly from controls (Figure 2B, *p* < 0.01). Although the levels of IgG1 and IgG2b were comparable to the infected control mice, significantly higher levels of IgG2a (*p* < 0.05) were observed in these animals and correlated with the partial protection observed in liver at 2 months postinfection. Interestingly, the IgG2a:IgG1 ratios of alum + LAg (0.96) and saponin + LAg (1.24) observed at 2 months post-infection maintained a bias towards Th2 and Th1 respectively, in keeping with our observations from sera obtained prior to *L. donovani* challenge. In contrast, mice vaccinated with lip + LAg exhibited higher levels of IgG2a and IgG2b, and a higher IgG2a:IgG1 ratio (1.47) than controls, strongly indicative of Th1 skewing.

With progressive infection at 4 months, both nonspecific and LAg-specific IgG levels were elevated in all groups including the PBS vaccinated and free-LAg vaccinated controls, however there was no significant difference in the nonspecific response within the LAg + adjuvanted groups (Figure 2C, *inset*). Moreover, we did observe that alum + LAg immunized mice showed higher levels of LAg-specific IgG1 (*p* < 0.05) and comparable levels of IgG2a to controls, culminating in a lower IgG2a:IgG1 ratio (0.8) (Figure 2C). Saponin + LAg immunization induced a trend of elevated IgG1 and IgG2a but the levels were not significantly different from the controls. However, saponin + LAg immunized mice nevertheless exhibited a high IgG2a:IgG1 ratio (1.12) reflecting stimulation of a Th1 biased immune response. In lip + LAg immunized mice the levels of IgG2a and IgG2b were again higher (*p* < 0.05) in comparison to both PBS and free adjuvant-immunized controls and showed a strong Th1 bias with a high IgG2a:IgG1 ratio (1.64), in keeping with the trend seen in this group post-vaccination.

The results thus demonstrate that although a nonspecific polyclonal antibody response is induced by *L. donovani* infection, there is no evidence that such a response influences the failure of protection or exacerbation of infection in alum + LAg or saponin + LAg conditions respectively. In contrast, higher levels of LAg-specific IgG1 and comparable levels of IgG2a in alum + LAg immunized mice indicated a Th2 bias, and correlated with an observed failure of protection in these animals. Although an inability to maintain high levels of IgG2a was observed in saponin + LAg immunized mice, the ratio of IgG2a:IgG1 nonetheless suggests a Th1 bias is extant post-immunization, that could be maintained 2 and 4 months post-infection. However, mice in this group not only failed to show protection in liver, but also exhibited exacerbation of infection in spleen. Only mice immunized with lip + LAg, showing elevated levels of both IgG2a and IgG2b, and exhibiting a high IgG2a:IgG1 ratio indicative of a strong Th1 bias, were protected during *L. donovani* challenge.

### Delayed type hypersensitivity (DTH) responses correlate with failure of protection but do not explain exacerbation of infection in immunized mice

**Figure 3:**
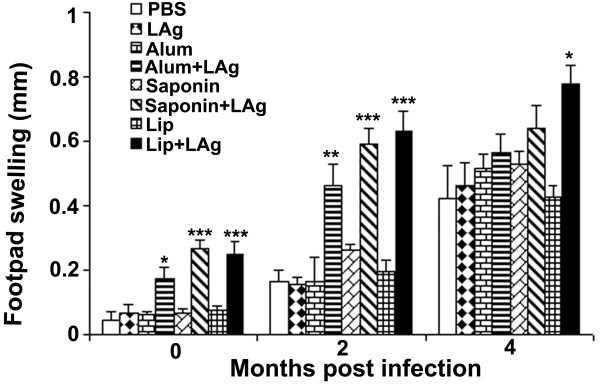
**DTH responses in vaccinated mice following immunization and *L. donovani *challenge infection.** LAg-specific DTH responses were measured ten days post-vaccination, or 2 and 4 months after challenge infection. DTH response is expressed as the difference (in millimeters) between the thickness of the test (LAg-injected) and control (PBS-injected) footpads at 24 h. Bars represent the mean ± SE of five individual mice per group, and are representative of two independent experiments. * *p* < 0.05, ** *p* < 0.01, *** *p* < 0.001 in comparison to PBS as well as free adjuvant immunized groups as assessed by one-way ANOVA and Tukey’s multiple comparison test.

To evaluate cell-mediated immune responses to LAg following vaccination, we monitored delayed-type hypersensitivity (DTH) responses in mice 10 days post-vaccination and 2 and 4 months post *L. donovani* challenge infection. Vaccination of mice with LAg in association with alum, saponin and liposomes all increased the DTH response (Figure 3, *p* < 0.05 in comparison to PBS as well as free adjuvant-immunized controls), and in addition at 2 months post- *L. donovani* challenge the response was further elevated in all of the vaccinated groups. The highest DTH response correlated well with the protection in lip + LAg immunized mice. We observed a partial reduction in parasite burden in liver after 2 months in alum + LAg and saponin + LAg immunized groups (Figure 1), which also correlated with high DTH responses induced in these animals (p < 0.01 in comparison to PBS as well as free adjuvant-immunized controls). However, at 4 months of infection mice immunized with alum + LAg and saponin + LAg showed minimal differences in DTH response as compared with PBS as well as free adjuvant-immunized controls. In contrast, lip + LAg immunized mice maintained elevated DTH responses significantly higher than controls (*p* < 0.05). The ability to sustain DTH responses at 4 months postinfection can be correlated with the ability of lip + LAg, but not alum + LAg or saponin + LAg vaccinated groups to protect against *L. donovani* challenge infection. However, we found no evidence that the DTH responses could explain the exacerbation of *L. donovani* infection observed in spleen of mice immunized with saponin + LAg observed at 4 months.

### Cytokine response in LAg + adjuvant immunized mice is a correlate of the clinical outcome following *L. donovani* challenge

**Figure 4:**
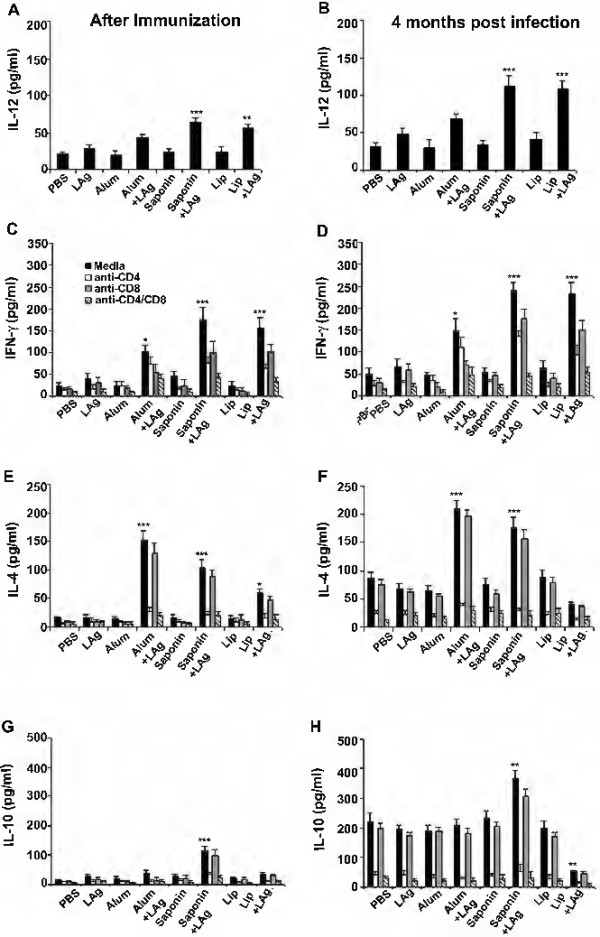
**Cytokine response in vaccinated mice following immunization and *L. donovani *challenge infection.** Ten days post-vaccination and 4 months after *L. donovani* challenge infection splenocytes were restimulated *in vitro* with LAg (10 μg/mL) in media alone or in the presence of anti-CD4 or anti-CD8 monoclonal antibody (1 μg/10^6^ cells). After 72 h supernatants were collected and assayed for IL-12 (**(A, B)**, IFN-γ **(C, D)**, IL-4 **(E, F)** and IL-10 **(G, H)**) by ELISA. Each sample was examined in duplicate. The results are shown as the mean ± SE for five individual mice per group, representative of two independent experiments with similar results. * *p* < 0.05, ** *p* < 0.01, *** *p* < 0.001 in comparison to PBS as well as free adjuvant immunized groups as assessed by one-way ANOVA and Tukey’s multiple comparison test.

Neither the humoral polyclonal antibody response nor the cell-mediated DTH response could entirely explain the observed disease progression in LAg + adjuvant immunized mice following challenge with *L. donovani*. We therefore asked whether LAg specific recall cytokine responses could provide use with a further mechanistic insight. To do so, we cultured splenocytes from experimental cohorts 10 days post-immunization, and 4 months after *L. donovani* challenge infection. Splenocytes from mice vaccinated with alum + LAg secreted significantly higher levels of IL-12 in comparison to free adjuvant-immunized controls (Figure 4A, *p* < 0.05). In addition, IFN-γ measured in splenocyte cultures was also significantly higher compared to both PBS and free adjuvant-immunized controls (Figure 4C, *p* < 0.05). We performed blocking experiments with anti-CD4 and anti-CD8 monoclonal antibodies to assess the relative contributions of CD4+ and CD8+ T cells to this cytokine production, revealing that IFN-γ secretion in alum + LAg immunized mice was produced mainly from CD8+ T cells, whereas CD4+ T-cell blocking had only a negligible effect. In contrast, the levels of IL-4 produced by CD4+ T cells was significantly higher not only in comparison to controls (Figure 4E, *p* < 0.001), but also to other remaining groups (*p* < 0.05). A low IFN-γ:IL-4 ratio (0.8) was observed in the alum + LAg vaccinated group and furthermore significant IL-10 production was not observed, remaining comparable to both PBS and free adjuvant-immunized controls (Figure 4G).

In contrast, splenocytes from saponin + LAg immunized mice produced significantly higher levels of IL-12 and IFN-γ in comparison controls (Figure 4A, C; *p* < 0.001). Notably, elevated levels of IL-4 and IL-10 were also produced by splenocytes of the saponin + LAg group (*p* < 0.001 compared to controls). Production of both IL-4 and IL-10 was substantially inhibited by addition of anti-CD4 blocking antibody to cultures, indicating that both of these cytokines were likely produced by the CD4+ T cell subset (Figure 4E, G). Despite exhibiting higher IL-4 and IL-10 levels following immunization, saponin + LAg immunized animals still exhibited high IFN-γ:IL-4 (1.59) and IFN-γ:IL-10 (1.60) ratios, perhaps demonstrating a subtle Th1 bias.

Finally, splenocytes from mice immunized with lip + LAg secreted higher levels of IL-12 and IFN-γ from both CD4+ and CD8+ T cells, in comparison to those immunized with PBS as well as free adjuvant immunized control groups (*p* < 0.01). Lip + LAg immunized mice additionally exhibited low although still statistically significant IL-4 production, secreted mainly from CD4+ T cells (*p* < 0.05 compared to controls), whereas IL-10 production was not observed in this group, above background.

We asked whether early cytokine production was indicative of subsequent outcome following *L. donovani* infection. Four months after *L. donovani* challenge, low levels of IL-12 (Figure 4B) and IFN-γ (Figure 4D) with elevated levels of IL-4 (Figure 4F) and IL-10 (Figure 4H) were observed in the culture supernatants of splenocytes of PBS and free adjuvant vaccinated control animals, as reported previously [6]. In alum + LAg immunized mice the level of IFN-γ, secreted mainly from CD8+ T cells, was elevated (*p* < 0.01 compared to both PBS and free adjuvant-immunized control groups). Although IL-10 levels remained comparable to controls, the levels of IL-4 produced in alum + LAg immunized mice were significantly enhanced at 4 months post-challenge infection (*p* < 0.001). Moreover, the IFN-γ:IL-4 ratio (0.74) remained low suggesting a Th2 bias in this condition.

In saponin + LAg vaccinated mice, we were surprised that IFN-γ secreted from both CD4+ and CD8+ T cells actually increased post-infection (*p* < 0.001 compared to controls), despite the failure of this vaccine regimen to induce protection. Moreover, the levels of IFN-γ measured in the splenocyte culture supernatants remained higher in comparison to alum + LAg immunized mice (*p* < 0.01). However, notably the CD4+ T cell derived IL-4 and IL-10 production was also significantly increased following saponin + LAg vaccination, showing elevation over both PBS as well as free adjuvant-immunized control groups controls (*p* < 0.01). Although a high IFN-γ:IL-4 ratio (1.34) was observed demonstrating Th1 bias, a low IFN-γ:IL-10 ratio (0.6) was found to correlate with the exacerbation of infection in spleen observed following *L. donovani* challenge (Figure 1).

Splenocytes of mice immunized with Lip + LAg showed enhanced production of IL-12 and IFN-γ at 4 months (*p* < 0.01) in comparison to controls, and our experiments showed that IFN-γ production occurred from both CD4+ and CD8+ cells (Figure 4B, D). Low levels of IL-4 and IL-10 secreted from CD4+ T cells were observed (*p* < 0.01 in comparison to controls) with a high IFN-γ:IL-4 (5.69) and IFN-γ:IL-10 (4.6) ratio also seen in this group (Figure 4F, H). The ratio implicated that a strong Th1 bias may be an important correlate of protection within this group.

In sum, we found that high IFN-γ and IL-12 production correlated with protective immunity following administration of a lip + LAg vaccine regimen. In contrast, despite the presence of elevated IFN-γ, the concurrent upregulation of IL-4 in alum + LAg immunized mice apparently overrode any protective effect exerted by IFN-γ, and correlated with failure of protection. Furthermore, high levels of both IL-4 and IL-10 correlated with exacerbation of disease in *L. donovani* challenged mice that had been vaccinated with saponin + LAg. These results clarify the differential immunological effects exerted by alternative adjuvants formulated with the LAg antigen and delivered subcutaneously.

## Discussion

Despite the fact that the majority of vaccines licensed for clinical use against VL remain live, attenuated, or killed crude preparations [2,3], much effort has been devoted to identify new *Leishmania* subunit/adjuvant combinations that are clinically efficacious. However, there are only few suitable adjuvants that have been licensed for human and veterinary vaccine use. Thus, a successful anti-leishmanial subunit vaccine will need to be assessed with human-compatible adjuvants. In our laboratory we have identified LAg as a potential candidate antigen, which was efficacious when associated with liposomes and vaccinated intraperitonealy in mice and hamsters [4,5]. However, In contrast to other reports utilizing differential liposomal formulations and administered subcutaneously [22,23], comparative evaluation of intraperitoneal and subcutaneous vaccination with LAg entrapped in our liposomal composition failed to protect against challenge infection through subcutaneous route [6]. Alum remains the most widely used adjuvant in human vaccines, and saponin is one of the promising adjuvant that has more recently been licensed for human use [7,12]. To facilitate broad clinical applicability, the preferred route of delivery is the minimally invasive subcutaneous route. Thus in an attempt to overcome the failure of subcutaneous vaccination with LAg in liposomes, this study investigated the protective ability of LAg in formulation with two widely used human-compatible adjuvants when injected subcutaneously.

Alum has been conventionally used as a clinical adjuvant for a wide range of vaccines that target a humoral immune response. However, the use of alum as an adjuvant for vaccination against the intracellular pathogen *Leishmania* has also been tested previously. In *L. major*, a vaccine containing killed parasites and IL-12 adjuvant was found to be prophylactically ineffective [24], however this antigen along with alum and IL-12 did induce protection in primates [8]. Moreover, encouraging results following vaccination in a primate model with combinations of alum-precipitated ALM and either BCG [9] or IL-12 [8] formed the basis of a human trial for a potential vaccine against VL. Safety and immunogenicity studies conducted under field conditions including healthy volunteers [10], as well as children who are at high risk of VL [25], indicated that the vaccine containing alum-precipitated ALM with BCG was safe and well tolerated. Again, the observation that the vaccine was highly immunogenic and could induce a strong Th1 response [10,26] led to the use of the formulation as an immunological stimulus for the successful treatment of patients with persistent PKDL [11]. Despite these satisfactory results, to our knowledge, such a formulation has not been examined for its efficacy in trials against VL. Herein we observed that alum + LAg failed to protect BALB/c mice against challenge with *L. donovani*. We therefore envisage that inclusion of a second Th1 promoting adjuvant such as IL-12 or BCG with alum will be necessary for an alum containing vaccine to be clinically successful against both CL and VL [8,9]. Nonetheless, it must be considered that failure of alum-ALM + BCG to protect susceptible BALB/c against *L. major*[27] raises some concern about the similar use of such an adjuvant in humans.

Saponin remains the immunopotentiator of choice in many cancer and infectious disease vaccine trials, such as malaria, HIV, hepatitis and tuberculosis [12]. In experimental VL FML or the immunodominant leishmanial antigen (NH36) formulated with saponin was found to be effective when administered prophylactically [13,28], and furthermore such formulations were also found to be efficacious when utilized immunotherapeutically [14,16]. These results facilitated the development of the currently licensed vaccine Leishmune®, composed of FML with increased amounts of saponin for field trials against canine VL. Indeed, Leishmune® has been recently shown immunotherapeutic potential for vaccination against canine VL [17]. In contrast to these reports, our study showed that saponin + LAg immunization not only failed to reduce parasite burden in liver of *L. donovani* challenged mice but also caused exacerbation of infection in spleen. These findings are partly in keeping with those of Grenfell *et al.*, who observed that antigenic extracts of *L. amazonensis* or *L. braziliensis* in association with saponin conferred only partial protection against *L. chagasi*[29]. Thus, the efficacy of saponin with leishmanial antigens other than FML may vary, and such observations warrant further pre-clinical studies to establish the potential of saponin to adjuvant vaccines against leishmaniasis.

Hypergammaglobulinemia and non-specific polyclonal antibody responses are hallmarks of VL. However, vaccine-induced antigen specific humoral response and their isotype profiles are often used as convenient surrogate markers of Th1 and Th2 response [21]. Evidence from both human patients and mice indicate that B-cell activation and production of polyclonal IgG may contribute to disease pathogenesis, leading to exacerbation of disease [19,20]. The absence of a detectable non-specific IgG response in mice immunized with alum + LAg and saponin + LAg suggests that polyclonal antibody responses do not contribute to the failure of protection in our system. Conversely, isotypic analysis revealed high levels of IgG1, IgG2a and IgG2b in both groups and demonstrate a mixed Th1/Th2 response. With infection the alum + LAg group failed to maintain the levels of IgG2a and IgG2b but nonetheless exhibited elevation of IgG1, reflecting a dominance of Th2, which correlates with the failure of protection in this group. In contrast, saponin + LAg immunized mice showed levels of IgG2a, IgG2b and IgG1 comparable with controls. Nevertheless, an increased IgG2a:IgG1 in the saponin + LAg condition is suggestive of a subtle Th1 bias, but it remains unclear how this may relate to the exacerbation of challenge infection in the spleen. Mice immunized with lip + LAg induced high levels of both IgG2a and IgG2b revealing that strong Th1 dominance is a correlate of protection in this group.

In an effort to further define the mechanism/s underlying protection induced by intraperitoneal lip + LAg versus the inability of subcutaneous immunization with alum + LAg or saponin + LAg to induce protection, we finally analyzed cytokine production by vaccinated cohorts in response to re-stimulation with LAg *in vitro*. Analysis of cytokines from splenocytes *ex vivo* revealed that animals vaccinated with lip + LAg produced high levels of both IL-12 and IFN-γ. Specifically we found that CD4+ and CD8+ T cells both contributed to this cytokine production, and may play an essential role in inducing resistance versus *L. donovani*[5,6,18]. Immunization with lip + LAg also enhanced the production of IL-4 and thus substantiated earlier observations from our lab and others suggesting that low levels of IL-4 at early time points are not detrimental and may even be beneficial in promoting Th1 differentiation, both maintaining IFN-γ production and priming IL-12 production in VL [5,18,30-32].

In contrast, mice vaccinated with alum + LAg produced low but nevertheless detectable levels of IFN-γ derived mainly from CD8+ T cells, whereas we also observed a robust IL-4 response from CD4+ T cells in these conditions. It is well established that alum promotes Th2 responses [7], but recently Serre *et al.* found that alum-precipitated proteins can also induce CD8+ T cells to produce Th1-associated IFN-γ [33]. In *L. major*, susceptibility to infection is related with the Th1/Th2 balance, and in particular IL-4 expression has been implicated as playing a role. Protective efficacy of vaccine formulations in CL is related not only with induction of Th1 responses but also the prevention of a Th2 response. Th2 responses have been suggested to override and thus abrogate even a strong Th1 effector function [34]. The higher levels of IL-4 induced by alum + LAg immunization in comparison to other vaccinated groups may therefore hinder the protective efficacy in this group. Thus, the failure of protection in alum + LAg immunized mice may be a direct result of the strong IL-4-driven Th2 response that predominated.

Interestingly, we observed that saponin + LAg immunized mice produced high levels of IL-12 and IFN-γ from both CD4+ and CD8+ T cells suggesting an overriding Th1-skewed response in this group. Such effects were also paralleled with significantly elevated Th2 cytokine production, namely IL-4 and IL-10, that was predominantly CD4+ T cell dependent. Several authors have shown an ability of saponin to upregulate the production of IFN-γ [12,13,28]. However, to our knowledge, our report represents the first observation that a saponin adjuvanted vaccine can induce robust IL-4. On the contrary, Greenfell *et al.*, reported that vaccination with antigenic extracts of *L. braziliensis* and *L. amazonensis* associated with saponin resulted in reduced production of IL-4 [29]. There are few reports of low levels of IL-10 production [35] and a low ratio of IFN-γ/IL-10 producing T cells [28] with vaccination of FML antigen or its component formulated with saponin in mice. However, most of the studies with these formulations have not been investigated for the stimulation of IL-10 production. In contrast, strong IL-10 as well as IL-4 responses was observed following immunization of *Trypanosoma cruzi* lysate adjuvanted with saponin [36]. Studies in humans [37], in mice with genetic ablation of IL-10 [38], or in conjunction with IL-10 receptor blockade [39], established that IL-10 is the major immunosuppressive cytokine in VL. The generalized negative regulatory role of IL-10 in vaccine failure is indeed well established [40]. Interestingly, exacerbation of *L. major* infection was associated with higher levels of both IL-4 and IL-10 relative to IFN-γ [41]. Consistent with this study, our results suggest that IL-10 is a major determinant of *L. donovani* disease progression in saponin + LAg vaccinated mice, and moreover IL-10 may collude with IL-4, to override the proinflammatory functions of IFN-γ.

*L. donovani* infection is characterized by distinct organ-specific pathogen/immune interactions, whereby the liver is the site of infectious resolution, whereas the spleen represents the site of parasitic persistence. In the liver, IFN-γ produced by both NK cells and T cells functions to resolve *L. donovani* infection [42]. In keeping with these findings, saponin + LAg immunized mice induced robust IFN-γ leading to specific protection in the liver at an early stage of infection (2 months). Infection models have produced unequivocal evidence that IL-10 is responsible for pathogen persistence [42,43] and thus, neutralization of IL-10 resulted in more effective clearance of *Leishmania* from the splenic compartment [44]. Thus, simultaneous production of high IL-4 and IL-10 may be the mechanistic determinant of the exacerbated infection observed in the spleen of saponin + LAg immunized mice.

Taken together, our study highlights the difficulties underlying the search for a highly efficacious leishmanial subunit vaccine in a clinical setting. The results herein support a model whereby efficacious subcutaneous vaccine formulations will be predicted to target both robust IFN-γ production and a strong Th1 response, but must minimally induce the immunosuppressive cytokines IL-4 and IL-10.

## Conclusions

Our data show that vaccination with alum + LAg and saponin + LAg failed to reduce hepatic parasite burden in BALB/c mice. Moreover, whereas alum + LAg immunization also led to vaccine failure as evidenced in the splenic compartment, saponin + LAg immunization actually resulted in exacerbation of *L. donovani* infection in this organ. A high IL-4 response coinciding with enhanced IgG1 correlated with a failure of protection in alum + LAg immunized mice, whereas exacerbation of infection in saponin + LAg immunized mice may involve the unbalanced secretion of IL-4 in conjunction with IL-10.

Critically, these results highlight that a limitation to administer LAg through the subcutaneous route cannot be overcome with the use of the human-compatible adjuvants alum or saponin, tested herein. Moreover, vaccines targeting *Leishmania*, should aim to generate robust IFN-γ, whilst preventing unfavourable increases of immunosuppressive cytokines including IL-4 and IL-10. We suggest that further detailed examination of the immunoregulatory responses governing IFN-γ, IL-4 and IL-10 production in immunized mice will greatly focus *a priori* design considerations necessary to speed production of novel leishmanial vaccines.

## Methods

### Animals

BALB/c mice were bred in the animal facility of Indian Institute of Chemical Biology Kolkata, India, and were between 4–6 weeks of age at the onset of the experiments. All animal studies were performed according to the Committee for the Purpose of Control and Supervision on Experimental Animals (CPCSEA), Ministry of Environment and Forest, Govt. of India, and approved by the animal ethics committee (147/1999/CPSCEA) of Indian Institute of Chemical Biology.

### Parasite culture

*L. donovani* strain AG83 (MHOM/IN/1983/AG83) was maintained by serial passage in hamsters and BALB/c mice as described elsewhere [4]. Promastigotes were grown and subcultured at 22°C in Medium 199 (pH 7.4) supplemented with 20% heat inactivated fetal bovine serum (FBS), 2 mM L-glutamine, 100 U/mL penicillin, 25 mM HEPES, 100 μg/ml streptomycin sulphate (all from Sigma-Aldrich, St. Louis, MO, USA). Subcultures were undertaken at an average density of 2 × 10^6^ cells/mL.

### Preparation of LAg and adjuvants

LAg was prepared from *L. donovani* promastigotes as described previously [4]. Briefly, stationary-phase promastigotes, harvested after the third or fourth passage, were washed three times in cold phosphate-buffered saline, pH 7.2 (PBS), pelleted and resuspended at a concentration of 20 mg/mL in cold 5 mM Tris–HCl buffer (pH 7.6). The suspension was centrifuged at 2,310 × *g* for 10 min to obtain crude ghost membrane pellet, resuspended in Tris–HCl buffer and sonicated for 3 min using an ultrasound probe sonicator (Misonix, Farmingdale, NY, USA). The suspension was clarified by centrifugation (5,190 × *g* for 30 min), and supernatant containing the LAg was stored at −70°C until use. The amount of protein obtained from a 1.0 g cell pellet was approximately 10 mg, as assayed by the method of Lowry *et al.*[45]. Imject alum purchased from Pierce (Pierce, Rockford, IL, USA) and saponin purchased from Sigma-Aldrich were used as adjuvants. Imject Alum was mixed with LAg diluted in PBS in a final ratio of 1:1. Saponin reconstituted at 1 mg/ml in PBS was injected at 20 μg/dose with LAg. Liposomes were prepared with egg lecithin (27 μmol), cholesterol, and stearylamine (Sigma-Aldrich) at a molar ratio of 7:2:2 as described previously [4]. Empty and LAg containing liposomes were prepared by the dispersion of lipid film in 1 ml PBS alone or containing 1 mg/ml LAg. The amount of associated LAg per milligram of egg lecithin was 36 μg.

### Immunization protocol and challenge infection

The experimental groups consisted of 4–6 weeks old BALB/c mice. Mice (5 mice per group) were immunized subcutaneously with 20 μg of LAg in PBS [4], either with alum or saponin in a total volume of 200 μl. Mice were boosted twice at 2 week intervals. Alternatively, mice were immunized three times with empty liposomes or 20 μg of LAg incorporated into liposomes, by intraperitoneal route, in a total volume of 200 μl at 2-week intervals. Ten days after the last immunization the animals were challenged with 2.5 × 10^7^ freshly transformed stationary phase *L. donovani* promastigotes in 200 μl PBS injected intravenously via the tail vein [4].

### Evaluation of infection

Two and 4 months post *L. donovani* challenge infection, cohorts of mice were monitored by the microscopic examination of Giemsa stained impression smears of liver and spleen. Parasite load was expressed in Leishman Donovan units, calculated by the following formula: number of amastigotes per 1,000 cell nuclei × organ weight (mg) [46].

### Assessment of delayed type hypersensitivity response (DTH)

Delayed type hypersensitivity (DTH) responses were evaluated by comparing the footpad swelling following intradermal inoculation with 50 μL of LAg (800 mg/mL) after 24 h relative to an alternative PBS control injection. Swelling was measured using a constant pressure caliper (Starrett Company, Athol, MA, USA) [4].

### Determination of antibody responses by ELISA

Sera from individual mice in each experimental group were collected before and after challenge with *L. donovani*. 96-well Microtiter plates (Maxisorp, Nunc, Roskilde, Denmark) were coated overnight at 4°C with either chicken egg albumin (OVA, Sigma–Aldrich, 25 μg/mL) or LAg (25 μg/mL) diluted in 0.02 M phosphate buffer (pH 7.5). Nonspecific binding was blocked with 1% bovine serum albumin in PBS, and the plates were subsequently washed with PBS containing 0.05% Tween 20. To measure total IgG, plates incubated overnight at 4°C with mouse sera were incubated for 3 h with polyclonal goat anti-mouse IgG conjugated to HRP (Sigma-Aldrich). To measure IgG1, IgG2a and IgG2b, plates were incubated overnight with monoclonal goat anti-mouse IgG1, IgG2a and IgG2b (Sigma-Aldrich) followed by HRP conjugated rabbit anti-goat IgG (Sigma-Aldrich) for 3 h. Wells were washed with PBS and incubated for 30 min with o-phenylenediamine dihydrochloride (0.8 mg/ml in 0.05 M phosphate citrate buffer, pH 5.0, containing 0.04% H_2_O_2_). Finally, absorbance was determined at 450 nm in an ELISA plate reader (Thermo, Waltham, MA, USA).

### Cytokine assays

Single cell suspensions of splenocytes were prepared in RPMI 1640 supplemented with 10% FBS, l00 U/mL penicillin G sodium, 100 μg/mL streptomycin sulfate and 50 μM β-mercaptoethanol (Sigma-Aldrich) (complete medium). RBCs were lysed with 0.14 M Tris buffered NH_4_Cl, and the remaining cells were washed twice with complete medium. Viable mononuclear cell numbers were determined with a hemocytometer. Cells were cultured in triplicate in a 96-well flat bottom plate (Nunc) at a density of 2 × 10^5^ cells/well in a final volume of 200 μL complete medium and stimulated with LAg (10 μg/mL) in media alone or in the presence of anti-CD4 and anti-CD8 monoclonal antibodies (1 μg/10^6^ cells; BD Pharmingen, San Diego, CA, USA). After 72 h incubation, culture supernatants were collected and the concentration of IL-12, IFN-γ, IL-4 and IL-10 (BD Pharmingen) was quantitated by ELISA in accordance with the manufacturer’s instructions and as described previously [6].

### Statistical analysis

One-way ANOVA statistical test was performed to assess the differences among various groups. Multiple comparisons Tukey-Kramer test was used to compare the means of different experimental groups. A value of *P* < 0.05 was considered to be significant.

## Competing interests

The authors declare that they have no competing interests.

## Authors’ contributions

Conceived and designed the experiments: SB, RR, NA. Performed the experiments: SB, RR. Analyzed the data: SB, RR, NA. Contributed reagents/materials/analysis tools: SB, RR, NA. Wrote the paper: SB, NA. All authors read and approved the final manuscript.

## Authors’ information

NA, Ph.D., Chief Scientist (CSIR), Infectious Diseases and Immunology Division, Indian Institute of Chemical Biology, Kolkata, West Bengal, India; SB, Ph.D., Assistant Professor, Department of Zoology, Dr. Kanailal Bhattacharyya College, Dharmatala, Ramrajatala, Santragachi, Howrah-711104, India; RR, Ph.D., Department of Pathology, Emory Vaccine Center, 954 Gatewood Road, Atlanta, GA 30329, USA.
